# Improving Drought Stress Tolerance in Ramie (*Boehmeria nivea* L.) Using Molecular Techniques

**DOI:** 10.3389/fpls.2022.911610

**Published:** 2022-06-30

**Authors:** Adnan Rasheed, Yucheng Jie, Muhammad Nawaz, Hongdong Jie, Yushen Ma, Adnan Noor Shah, Muhammad Umair Hassan, Syed Faheem Anjum Gillani, Maria Batool, Muhammad Talha Aslam, Ahmad Raza Naseem, Sameer H. Qari

**Affiliations:** ^1^College of Agronomy, Hunan Agricultural University, Changsha, China; ^2^Department of Agricultural Engineering, Khwaja Fareed University of Engineering and Information Technology, Rahim Yar Khan, Pakistan; ^3^Research Center on Ecological Sciences, Jiangxi Agricultural University, Nanchang, China; ^4^College of Agronomy, Gansu Agricultural University, Lanzhou, China; ^5^College of Plant Science and Technology, Huazhong Agricultural University, Wuhan, China; ^6^Department of Agronomy, University of Agriculture, Faisalabad, Pakistan; ^7^Institute of Soil and Environmental Sciences, University of Agriculture, Faisalabad, Pakistan; ^8^Department of Biology, Al-Jumum University College, Umm Al-Qura University, Makkah, Saudi Arabia

**Keywords:** ramie, drought, yield, genes, marker-assisted-selection, CRISPR/Cas9

## Abstract

Ramie is one of the most significant fiber crops and contributes to good quality fiber. Drought stress (DS) is one of the most devastating abiotic factors which is accountable for a substantial loss in crop growth and production and disturbing sustainable crop production. DS impairs growth, plant water relation, and nutrient uptake. Ramie has evolved a series of defense responses to cope with DS. There are numerous genes regulating the drought tolerance (DT) mechanism in ramie. The morphological and physiological mechanism of DT is well-studied; however, modified methods would be more effective. The use of novel genome editing tools like clustered regularly interspaced short palindromic repeats (CRISPR) is being used to edit the recessive genes in crops to modify their function. The transgenic approaches are used to develop several drought-tolerant varieties in ramie, and further identification of tolerant genes is needed for an effective breeding plan. Quantitative trait loci (QTLs) mapping, transcription factors (TFs) and speed breeding are highly studied techniques, and these would lead to the development of drought-resilient ramie cultivars. The use of hormones in enhancing crop growth and development under water scarcity circumstances is critical; however, using different concentrations and testing genotypes in changing environments would be helpful to sort the tolerant genotypes. Since plants use various ways to counter DS, investigating mechanisms of DT in plants will lead to improved DT in ramie. This critical review summarized the recent advancements on DT in ramie using novel molecular techniques. This information would help ramie breeders to conduct research studies and develop drought tolerant ramie cultivars.

## Introduction

Abiotic stresses are causing a huge decline in crop growth and production worldwide (Fahad and Bano, 2012; Fahad et al., 2013, 2017, 2019a, Fahad et al., 2021c, Bamagoos et al., 2021). Drought stress (DS) is the worst environmental factor around the globe and severely affects crop yield (Anjum et al., 2011; Alharby and Fahad, 2020; Seleiman et al., 2021; Yahaya and Shimelis, 2022). DS is an inevitable abiotic aspect in numerous ecological areas, severely declining crop yield and quality without warning before its onset (Seleiman et al., 2021; Bandurska, 2022). Low rainfall, temperature, and light intensity contribute to drought incidents (Zia et al., 2021). DS causes the oxidation of the cell by the generation of reactive oxygen species (ROS) (An et al., 2015a). ROS at a definite threshold in plant tissues degrade the biological membrane system, and therefore, the cell ultrastructure is impaired (Hassan et al., 2017; Chattha et al., 2021; Hassan et al., 2021; Shah et al., 2021; Batool et al., 2022a; Jinhua et al., 2022; Rehman et al., 2022). DS tolerance comprises molecular, morphological, and physiological paths, including introducing and silencing numerous genes and improving antioxidants action (Aslam et al., 2022). Plants have evolved a variety of complex networks against DS (An et al., 2015b, 2016).

**FIGURE 1:**
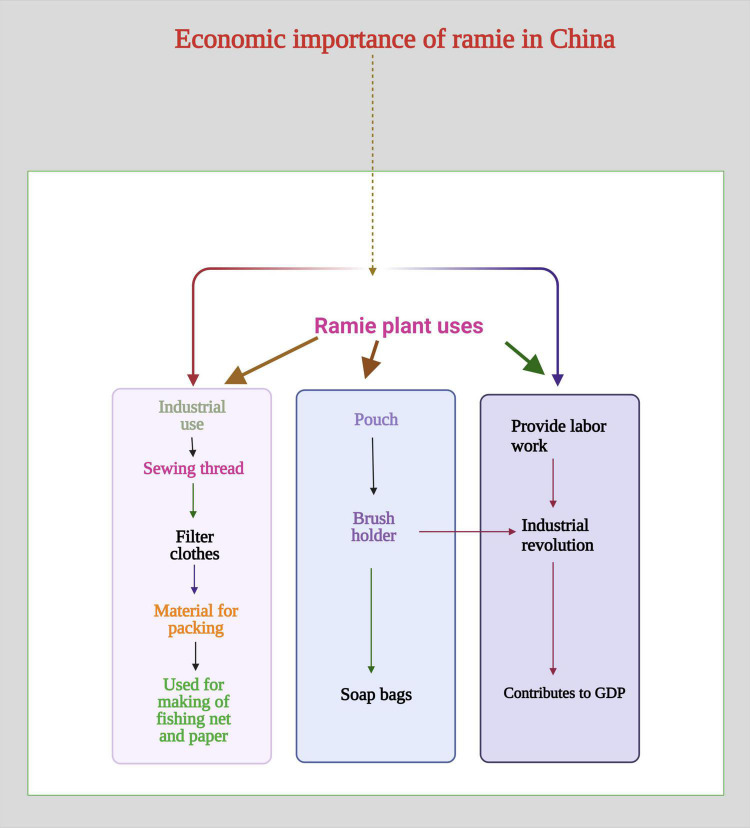
Economic importance of ramie in China. Ramie is used for making sewing thread, filter clothes, packing material, fishing net, and paper. Ramie is also used for making pouch, brush holder, and soap bags. It provides labor work and has a significant contribution to gross domestic product (GDP).

Ramie is one of China’s most famous fiber crops with excellent fiber quality as shown in Figure 1 (Rehman et al., 2020, 2021). As it used for making products like packing material, filter clothes, fishing nets, and soap bags (Figure 1). Ramie is an important economic crop in China (An et al., 2015b, 2016; Wu et al., 2021a). Ramie is mainly cultivated in China under outdated farming methods. The production and success of ramie farming systems are deteriorating slowly, and there is a critical need to improve farming methods. Because of this reason, the need to identify genetic factors behind drought tolerance (DT) is a critical need at this time (Satya et al., 2019). There is an urgent need to investigate different signaling cascades because of DS, keeping in mind the current situation to identify the genetics of DT mechanism in ramie (An et al., 2015b). Plants have developed two types of response against DS, the short-term and long-term response (Tardieu, 2022).

The long-term response includes abortion of grains, completing life cycle, allocating nutrients, and delayed leaf senescence mechanism (Chen et al., 2016). Stomatal conductance, water potential differences, osmolyte content, and maintenance of turgor pressure are related to short-term responses (Zahedi et al., 2022). DS reduces water potential, as a result, plants accumulate more solutes in the cytosol and other cell compartments. Therefore, drought disturbs the growth of the crops and their reproductive phase at the whole plant level (Sharma and Sardana, 2022). DS induces numerous fluctuations at morphological, biochemical, and physiological stages, and it is categorized by a reduction in leaf water potential and reduced cell expansion (Chen L. et al., 2021). DS causes abnormal starch accumulation, which affects the viability of pollen (Lamin-Samu et al., 2021), and rolling of leaf, shadowed by wilting and decolorizing that eventually leads to the plant’s death (Seleiman et al., 2021). Plant researchers witnessed that water shortage in roots is due to the dryness of soils while in the leaf cells it is due to tiny air moisture and higher temperatures (Ozturk et al., 2021). DS also leads to reduced leaf area (Ozturk et al., 2021), decreased mitosis, cell enlargement, and cell growth. Main alterations in leaf anatomy and structure like leaf size reduction and stomatal reduction are studied in many crops (Ilyas et al., 2021). Noteworthy development has been made to understand the plant’s reaction to DS at various levels (Ahmad et al., 2021).

Any stressful situation causes differences in gene functions, bringing variations in the arrangement of the plant transcriptomes, metabolites, and proteome (Singh et al., 2021). Molecular techniques like, CRISPR/Cas9 and speed breeding can play a key role in development of tolerant ramie cultivars and need further studies to expand their use. CRISPR/Cas9 can cause targeted gene editing in ramie and speed breeding is used to develop the cultivars in short period of time. As genes and QTL play a vital role in signaling development, transcription regulation, detoxification at the cellular level, safeguarding of macromolecules, and a collection of other cellular procedures, numerous genetic tools have now been developed that are helpful in recognizing the mechanisms linked with DT in crops (Bharadwaj et al., 2021). Thus, while the development of drought-tolerant ramie crops is practically important, only a few reports have focused on its genetic improvement. A few studies have reported on the physiological and molecular factors (Liu et al., 2013b) controlling DT in ramie. Earlier studies recognized 24 transcription factors (TFs) that can be transcripts, but only 12 are potentially involved in DT (Liu et al., 2013c). The combined use of different breeding tools is critical in improving ramie growth and yield under water deficit conditions (Huang et al., 2012).

Different plant mineral nutrients like potassium (K) play a crucial role in plants under DS (Agarwal et al., 2006). K is actively involved in various pathways like enzyme activation, stomatal movement, and osmoregulation (Wang et al., 2013). K encourages root growth during water stress conditions because it increases sucrose transport to the young roots for their average gain. K also increases the uptake of ions as it is one of the main constituents of phloem sap. Therefore, it mitigates the adverse effects of DS by maintaining the water balance within plants (Ahanger et al., 2014). Secondly, nitrogen (N) which is a vital plant nutrient required in significant quantities by plants, also improves DS tolérance (Liu et al., 2008). Studies have shown that appropriate N supply in plants significantly increased DT (Liu et al., 2008). Likewise, phosphorus (P) also contributes to DT when available in proper quantity. It maintains leaf water content, photosynthetic rate, and quantum efficiency under DS (Tariq et al., 2017). Hence, adequate plant nutrition can mitigate the adverse effects of DS. The adverse consequences of drought on numerous other crops were studied (Fahad et al., 2020, 2021f). Hence, it is critical need to develop climate resilient crops. There is a little information available on DT in ramie because of the lack of novel germplasm and detailed research studies. The aim of this review is to present a comprehensive overview of DS tolerance in ramie at various levels using several techniques. These techniques could play a key role in DT in ramie. In this review we have briefly discussed the conventional approaches, as well as molecular approaches and their role in the improvement of DT in ramie. This information would be of great significance in directing future research studies for breeding drought-tolerant ramie cultivars.

## Effects of Drought Stress on Ramie

**FIGURE 2:**
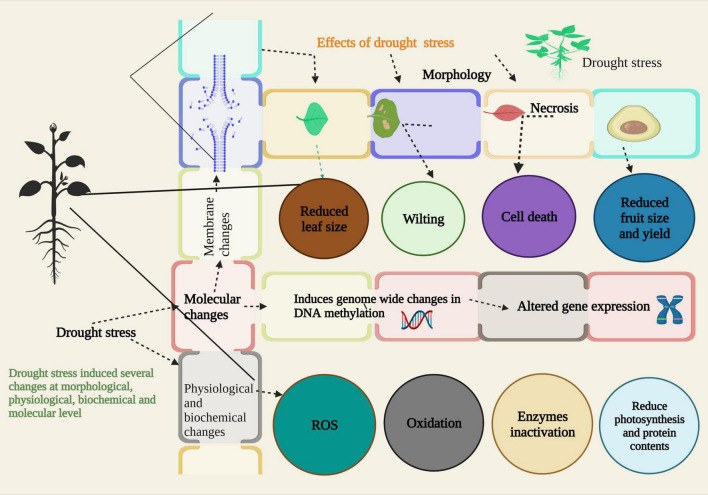
Effects of drought stress (DS) on various stages of plant growth and development. At morphological level, DS induces several changes in plant like, chlorosis, necrosis, wilting, reduced leaf size, cell death, and yield reduction. At physiological and biochemical level, DS also causes oxidation by production of reactive oxygen species (ROS), inactivation of enzymatic action, and reduced photosynthesis and protein contents. DS also brings certain alterations at molecular level like, changes in DNA methylation and altered gene expression.

DS has detrimental effects on crop growth and development. It affects the germination of seeds, growth, photosynthesis, stomatal conductance, plant–water relations, and yield (Saud et al., 2014, 2016, 2020; Figure 2). In the United States of America (USA), about 66% of plant yield was reduced because of prolonged DS (Comas et al., 2013). DS is one of the most devastating factors in arid and semi-arid areas. DS affects all phases of the plant directly or indirectly (Liu et al., 2022). DS reduced the concentration of humidity in soil which is necessary for seed germination. DS caused the late emergence of seedlings in ramie (Huang et al., 2013). Loss of turgor pressure, gas exchange, and oxidative damage are triggered by DS (Hussain et al., 2018). Drought episodes induce necrosis (Figure 2) due to overproduction of cells, reduced surface area of the leaf, and damage to several enzymatic actions (Dong et al., 2020). Drought-induced ROS causes oxidation, which may be useful or harmful for plants depending on their concentration (Challabathula et al., 2022). DS severely affected the uptake of plant nutrients which are essential elements in plant growth and development (Alam et al., 2021). Mineral composition, proteins, and antioxidants are affected by DS (Kosar et al., 2021; Figure 2). DS also causes genome-wide changes in DNA methylation and altered gene expression (Ackah et al., 2022). It also disturbs the enzymatic activity in the cell and affects many pathways (Shawon et al., 2020). Longtao et al. (2016) investigated the effect of DS on the physiology and yield of ramie cultivars, and they noted that DS significantly decreased the biomass production of cultivars. In another study, the effect of DS was studied on ramie cultivars, and results showed that the amount of malondialdehyde decreased as a result of DS (He et al., 2015). Ramie cultivars showed a severe decline in stem growth and fiber production under extreme DS (An et al., 2015a). Chlorophyll content of ramie also decreased under DS (Liu et al., 2013c). Likewise, Huang et al. (2012) investigated the effect of different drought episodes on chlorophyll content and showed that DS causes a decline in chlorophyll content. Response of different ramie cultivars showed that DS reduced the fiber length and quality in ramie (Liu et al., 2005; Table 1). Nutrients play an important part in crop growth and development. Disturbance in nutrient pathways led to severe decline in yield of ramie. It is obvious from earlier studies that DS affects the nutrient uptake in ramie cultivars. DS decreased the uptake of different plant nutrients and caused a decline in the crop’s yield (Liu et al., 2000). Due to the economic significance of ramie, it is now critical to mitigate the harmful effects of DS in order to stabilize the yield and quality. The impact of DS on ramie may vary with time and intensity of stress. To better understand the consequences of DS, it is essential to evaluate different ramie genotypes under other drought conditions and assess the effects at various growth stages of ramie. These studies will provide a better way to understand the consequences of DS and enable the development of a better mechanism for reducing the negative effects (Liu et al., 2005).

**TABLE 1:** Effects of DS on growth, yield and physiological traits of ramie crop.

Effects	References
Drought stress reduced the biomass production, plant height, growth rate, yield and activities of NR, and the activity of sucrose synthase	Longtao et al., 2016
Reduced activity of malondialdehyde	He et al., 2015
DS reduced the photosynthesis, relative water content (RWC), and increased activities of POD and proline contents	An et al., 2015b
Decrease in chlorophyll a and carotenoid contents	Liu et al., 2013a
10.15% reduction in chlorophyll and protein contents	Huang et al., 2012
Reduced plant height and antioxidants activity	Chen et al., 2012
Reduced fiber length, erectness, and leaf surface area	Liu et al., 2005
Reduced nutrients concentration, photosynthetic activity, and nitratase activity in leaves	Liu et al., 2000

## Drought Tolerance Mechanism in Ramie

**FIGURE 3:**
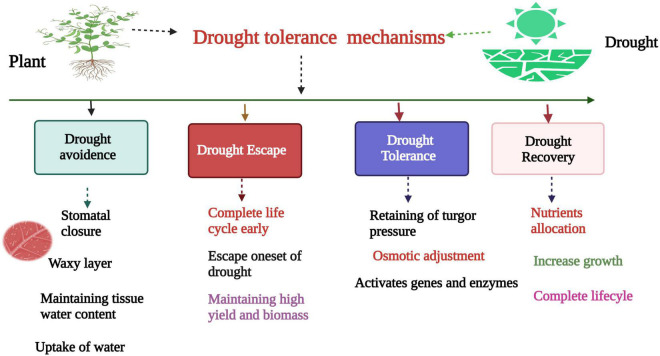
Drought tolerance mechanism in ramie. The drought tolerance mechanism involves four steps, DA, DE, DT, and DR. Drought avoidance involves, stomatal closure, waxy layer, maintaining water contents in tissues, and enhance water uptake. Drought escape involves the early completion of life cycle before initiation of drought episodes. Likewise, drought tolerance involves, osmotic adjustment, and genes and enzymes activation. Last step is drought recovery which involves the improvement of growth due to allocation of nutrients and complete life cycle.

Drought tolerance is a complex polygenic trait which involves a number of mechanisms and genes controlling this trait. Most of the genes have minor effects for DT. The complex nature of DT makes it difficult to improve the cultivars without a complete understanding of gene networks (Thanmalagan et al., 2022). Plants adopt several mechanisms to cope with DS (Fang and Xiong, 2015; Muiruri et al., 2021; Panda et al., 2021). These mechanisms include DT, avoidance, escape, and recovery after drought (Figure 3). Drought avoidance mechanisms include maintaining higher water content in tissue and normal functioning of physiological processes despite low water content in soil (Luo, 2010; Varshney et al., 2021). The plant closes its stomata, limits vegetative growth, increases water and nutrient uptake and waxy accumulation. In the drought escape mechanism, the plant completes its life cycle before one set of droughts. In DT, plants maintain turgor pressure and activate several genes and enzymes (Figure 3) which protect plants from detrimental effects of DS (Coussement et al., 2021). The ability to recover after severe DS is called drought recovery (Fang and Xiong, 2015; Giordano et al., 2021; Sun et al., 2021). Hence, DT is a complex polygenic trait, and it is essential to understand the factors behind it (Tripathi et al., 2022). Different genes are activated under DS and control different pathways to protect plants from detrimental effects. Different genes have been identified which confer DT and improve plant growth and development (Huang et al., 2016b). Several DT indicators like osmotic adjustment, leaf water potential, and proline content play a key role in DT (Fang and Xiong, 2015). Likewise, antioxidants and osmoprotectants are essential factors in developing drought-tolerant genotypes (Luo, 2010). In some studies, leaf morphology is also used as an indicator for developing drought-tolerant cultivars (Bogale et al., 2011). Earlier studies confirmed that genotypes with rolled leaves could reduce water loss under severe DS (Xiang et al., 2012). Reducing the transpiration rate is very critical for plants under DS. Under DS, the leaf starts wilting and, therefore, suppresses growth and development (Fanizza and Ricciardi, 2015). Plants adopt a thick waxy cuticle layer to cope with DS (Ullah et al., 2017). Leaves develop a smaller number of stomata, vascular tissues, and thick palisades (Iqbal et al., 2013). Recent studies have shown that overexpression of *MtCAS31* increased DT in *Arabidopsis* by dropping stomatal thickness (Xie et al., 2012). Some drought tolerant ramie cultivars (Zhongzhu No2, Zhongzhu 1) showed better growth under DS conditions (Liu et al., 2013a; Longtao et al., 2016). These cultivars can be used to transfer drought tolerant genes in sensitive cultivars. Further studies are required to investigate the in-depth analysis of plant response to DS. Plant response to DS at a molecular level needs further study to identify the number of genes controlling DT in ramie and their complex pathways.

## Agronomic Approaches to Enhance Drought Tolerance in Ramie

Application of crop management practices can potentially alleviate the harmful effects of abiotic stresses particularly DS (Fahad et al., 2015d, 2019b, 2021a,b; Wu et al., 2020). These agronomic approaches include, the use of certain plant hormones, and macro and micronutrients (Fahad et al., 2021d,e). These practices are widely used to maintain the growth and yield of crops under the changing environmental conditions (Yang et al., 2017; Zafar-Ul-Hye et al., 2020a,b, 2021).

## Use of Plant Growth Hormones and Plant Nutrients to Enhance Drought Tolerance in Ramie

**FIGURE 4:**
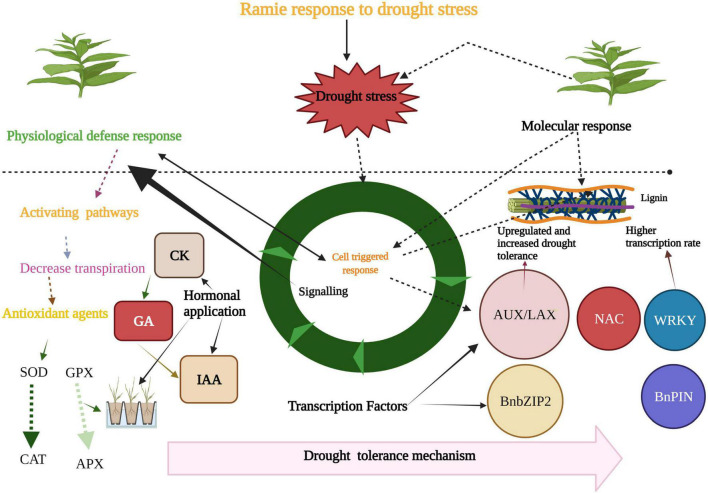
Role of different factors to enhance drought tolerance in ramie. Ramie response to drought stress (DS) by various ways like, activation of different genes which provide resistance to stress. Different antioxidants like SOD, GPX, APX, and CAT are used to counter the adverse effects of DS. Hormones like, GA, CK, and IAA are being used for mitigating the effects of DS.

The hormones play a crucial role in triggering defense responses in plants against several environmental factors (Fahad et al., 2015a,b, 2016a,b; Wu et al., 2019). These hormones mitigate the adverse effects of abiotic stresses and maintain crop growth and production (Fahad et al., 2015c, 2016c,d, 2018). Abscisic acid (ABA) is the primary hormone that enhances DT in crops by inducing several morphological and physiological changes. These changes include stomatal regulation, root development, and initiation of ABA-dependent pathways development. Besides this, salicylic acid (SA), auxin, and cytokinin’s (CK) also play a vital role in mitigating DS (Figure 4). CK enhanced the capacity of plants to tolerate water deficit conditions by improving the process of carbon assimilation and N metabolism (Reguera et al., 2013). Likewise, SA regulates the plant antioxidant defense system, stomatal movement, and photosynthetic rate under DS (Nazar et al., 2015). These hormones often crosstalk with each other to increase the DS tolerance in crops (Ullah et al., 2018). Meanwhile, different types of antioxidant enzymes like, superoxide dismutase (SOD), catalasa (CAT), ascorbate peroxidate (APX), and glutathione peroxidase (GPX) reduce the level of superoxide and hydrogen per oxide in cell (Figure 4). These are the most important enzymes used against oxidative stress and occur ubiquitously in all types of plant cells (Laxa et al., 2019). Osmolytes have been used to mitigate the adverse effects of DS in many crops. Osmolytes are small and highly soluble organic compounds which maintain osmotic balance under drought conditions. Plants overexpress osmolyte biosynthesis genes which enhances DT (Nahar et al., 2016). These osmolytes play a role in stabilizing proteins, scavenging of ROS, and balancing cellular redox under DS (Hasanuzzaman et al., 2019). The ramie genotypes were evaluated under the DS conditions. The treatments included severe DS, severe DS and GA3 spray, regular water, and GA3 spray, and normal water as a control. Results showed that proline contents and soluble sugar contents were increased in the ramie group exposed to DS and GA3 treatment. These outcomes recommended that exogenous GA3 can improve the DT in ramie (Liu et al., 2013a). Likewise, the effect of SA was studied on drought-affected ramie cultivar Zhongzhu No2. Exogenous SA was sprayed, and four treatments were applied. Results showed that sugar and protein contents were increased after the application of SA in DS conditions. The activities of three key enzymes responsible for fiber development were decreased as compared to the control on the 30th day of treatment. The exogenous application of SA recovered plant height and yield, but aerial biomass was reduced. Consequently, these results showed SA improved the actions of enzymes and alleviated the consequences of DS in ramie. This provides a theoretical foundation for DT in ramie (Longtao et al., 2016). Betaine plays a vital role in mitigating the harmful effects of DS in ramie. Huazhu No. 5 was grown to study the impact of exogenous betaine spray on the growth and yield of ramie grown under DS. The results suggested that spraying betaine could improve DT by lowering the relative conductivity ratio of ramie and increasing the activity of peroxidase (POD) at the growth stage. Betaine improved the fresh stem weight and bast weight compared to the control. These findings provided a solid theoretical basis for developing drought-tolerant ramie cultivars (He et al., 2015).

## Use of Nitrogen to Improve Drought Tolerance in Ramie

DS adversely affects the growth and development of ramie (Huang et al., 2012), which is one of the most significant fiber crops of China. Under dry conditions, crop growth is restricted due to decreased water availability (Asghar and Bashir, 2020). Different mineral nutrients also play a crucial role in improving DS tolerance in plants (Waraich et al., 2012). To reduce the lethal effects of stress, N fertilizer has been used to improve plant growth (Wajid et al., 2017). N deficiency in plants causes a decline in biomass production (Gong et al., 2019). Efficient N application can be used under DS (Shangguan et al., 2000; Saud et al., 2017; Mahpara et al., 2019). Earlier studies showed that shoot biomass is more affected by DS, and root biomass is less affected (Song et al., 2010). The availability of good soil N makes plants more drought-hardy than soil with a N deficiency (Halvorson and Reule, 1994). Increased N application improved crop growth and development. N minimizes the risk of plasma membrane damage and also maintains osmotic adjustment. It also leads to the increase in uptake of several plant nutrients like calcium (Ca) and K (Ahanger et al., 2017). N application reduced the content of malondialdehyde, and alleviates DS in crops (Saneoka et al., 2004). It recovers the injuries caused by DS and enhances the cell division rate, leading to an increase in leaf area (Wu et al., 2008). DS dramatically influences photosynthesis in crops, which is recovered by sufficient N application (Hammad et al., 2017; Hammad et al., 2020a,b). Hence, DT can be improved by the proper application of N. Therefore, it is critical need of time to evaluate the ramie genotypes under DS conditions and study the role of N application in mediation of growth and development.

## Use of Phosphorus to Improve Drought Tolerance in Ramie

Under DS conditions, plants cope with DS and maintain their growth and development by increasing photosynthesis rate and stomatal conductivity (Waraich et al., 2011a). Previously, many studies reported that P is an important plant nutrient (Zia et al., 2017; Wahid et al., 2020), and the use of a P dose under DS conditions significantly improved crop growth and development by enhancing their water usage efficiency (Waraich et al., 2011b; Hansel et al., 2017). The accessibility of optimum P in crops promotes growth by improving root growth and stomatal activity (Naeem and Khan, 2009). It optimizes the leaf area (Singh et al., 2013), enhances plasma membrane stability and water use efficiency (WUE) (Gislén et al., 2003; Faustino et al., 2013). Studies have shown that plants have higher P levels under drought conditions than plants grown under normal conditions, which indicate its role in DT in plants (Hansel et al., 2017). N mobility is also increased by P application under DS (Zhou et al., 2015). P application also improves several morphological and physiological traits like plant height, leaf area, and WUE (Singh et al., 2006). Previous studies have shown that the deep application method of P works effectively to mitigate the adverse effects of DS (Kang et al., 2014). This evidence strongly suggests the potential role of P to minimize the negative impact of DS in crops. Therefore, it is critical need of time to evaluate the ramie genotypes under DS conditions and study the role of P application in mediation of growth and development.

## Use of Potassium to Improve Drought Tolerance in Ramie

Potassium is an essential plant nutrient and holds the key for numerous physiological mechanisms like photosynthesis, protein synthesis, and osmoregulation (Zörb et al., 2014; Zahoor et al., 2017a; Zhu et al., 2020). Studies have shown a good relationship between water and soil K. Hence, plants absorb K to improve their WUE (Zhu et al., 2020), and increase the tolerance level to withstand water deficit conditions. A K dose changes the carbon dioxide input process by maintaining the stomatal function, which mitigates the photo-assimilation restricted by DS (Farooq et al., 2009). K also controls the enzymes involved in carbohydrate metabolism to increase sucrose translocation. These processes are related to plant growth and development (Zahoor et al., 2017b). Likewise, adding K doses in drought-affected soil can be helpful for high-yield crops (Ahanger et al., 2014). K increased the sugar and proline content under water deficit conditions. On the other hand, activity of different enzymes involved in DT is enhanced by supplementation of K and its increased concentration in the cytoplasm (Kant et al., 2002). K enhanced membrane fluidity by maintaining the balance of unsaturated to saturated fatty acids in the membrane (Wilkinson et al., 2001). Studies have shown that the K dose also increased the quantity of several essential solutes like sugar, amino acids, and proline under water deficit conditions, which strongly contributed to osmotic adjustments in the plant (Ahanger et al., 2014). K application showed promising results under DS conditions. These results suggested that K can also be used to improve DT in ramie to maintain its growth and development.

## Use of Micro Nutrients to Enhance Drought Tolerance in Ramie

Micronutrients also play a crucial role in improving crop DT (Hassan et al., 2020). Application of micronutrients under DS enhanced the activities of several antioxidants like SOD, CAT, and GPX (Rahimizadeh et al., 2007). Sajedi et al. (2011) studied the effects of zinc (Zn), copper (Cu), and boron (B) on plant grown under DS. Results showed that with an increase in DS, the micronutrients improved the activity of antioxidants and the yield of plants (Sajedi et al., 2011). Zn, a critical micro nutrient, plays a crucial role in promoting plant growth and development. It increased proline content and maintains antioxidant membrane permeability and activity under DS (Babaeian et al., 2010).

Studies have shown that Zn application under DS increases leaf area, chlorophyll content, and stomatal conductance (Karim et al., 2012). Likewise, Zn application mitigated the adverse effects of DS and increased yield in the plant (Sultana et al., 2016). Boron plays a critical role in improving DT in crops. At a rate of 4 mg L^–1^, B was applied to study the growth of plants under DS. B improved the plant water status, chlorophyll content, and antioxidant activity. These findings revealed the promising role of B in improving DS tolerance in crops (Naeem et al., 2018). Bhatia et al. (2005) found that nickel (Ni) maintained the osmotic adjustment of crops under DS. Cobalt (Co) is a crucial plant nutrient that increases leaf tolerance to dehydration and decreases the wilting of leaves under DS. A total of 12 ppm of Co application under drought pressure increased several growth parameters and enhanced yield. The role of Co shows that crop growth and production can be increased under water deficit conditions (Gad et al., 2018). Likewise, selenium (Se) is being used to reduce the adverse effects of DS in many crops. Studies showed that Se application increased the concentration of osmoprotectants and antioxidants activities under DS (Rady et al., 2020). These results showed the potent role of micronutrients in plant growth and development under DS. Calcium and manganese (Mn) also played a significant role in DT in crops (Ghorbani et al., 2019; Hosseini et al., 2019). However, further studies are required to fully understand nutrients interaction in plants and their possible role under DS. It might be better to study the combined effects of different macro and micro nutrients to mitigate the adverse consequences of DS. Many factors should be considered while analyzing the role of nutrients under drought pressure, like growth stage, crop type, and fertilizer rate (Rasheed et al., 2020b). Being a significant fiber crop in China, ramie can be grown under severe DS to increase its growth and yield.

## Molecular Techniques to Enhance Drought Tolerance in Ramie

The development of ramie cultivars which are tolerant to environmental stresses is a critical need at this time (Wu et al., 2021b). Developing crop genotypes with improved agronomic traits that offer resistance to abiotic stresses has long been an international concern (Romero-Galindo et al., 2022). The first step is to adopt reasonable practices under changing environmental conditions. To sustain crop production under DS is a big challenge. Plants have numerous defense mechanisms to withstand DS at the morphological and molecular level (Agarwal et al., 2006). Breeders are now trying to develop genetic traits which improve DS tolerance while maintaining yield under crucial situations (El-Mouhamady et al., 2022). Genetic engineering (GE) and other molecular techniques can significantly enhance DT in ramie and other crops. In earlier times, conventional breeding methods encouraged plant growth under water stressed environments. These methods are very time-consuming and costly, and therefore the development of molecular markers played a crucial role in detecting the genetic variability in crops (Chen et al., 2022). Many QTLs have been detected in many crops, but their reliability and accuracy are often problematic (Xu et al., 2022). By keeping this in mind, the genetic modification method has proven very useful in developing drought-tolerant cultivars (An et al., 2015b). Novel techniques that can increase the plant response to DS have been successfully authenticated. Gene editing techniques are being used to bring novel variations of desired traits in the genome (Georges and Ray, 2017). There use of latest molecular techniques would open new windows of hope to improve ramie under DS conditions. The application of molecular techniques would bring significant results and offer a opportunity to identify the potent genes accountable for certain molecular pathways under DS.

## Complete Genome Sequencing to Identify the Candidate Genes for Drought Tolerance

Next-generation sequencing (NGS) has been expanded during the last 10 years and provided a cost-effective and reliable way of single-nucleotide polymorphism (SNP) screening and development of high-density genetic maps (Verma et al., 2015; Branham et al., 2017). The progress in genome sequencing has gained wide attention in molecular fields (Chen et al., 2013). The genomic data can be studied, and individual candidate genes can be selected for a particular function. This approach is very efficient in underlying the mechanism of different factors in a plant like DS. Whole-genome sequencing can help to engineer crops for identification of genes for several functions. NGS has made sequencing very easy and affordable. A lot of studies have focused on genome sequencing of fiber crops like cotton (Li et al., 2015) and ramie (Liu et al., 2018). The complete genome sequencing of ramie would help to develop drought-tolerant ramie cultivars. Liu et al. (2018) determined the genome sequence of ramie to explore the molecular basis for different traits, however, no study on DT has been found. Hence, a complete genome sequencing is required to identify the genetic factors regulating DT in ramie. Further studies are required which should focus on the identification and characterization of important genes regulating DT in ramie. Identification and cloning of genes would speed up the molecular breeding programs. The above studies set a new foundation for future use of this technique in ramie (Liu et al., 2018).

## Quantitative Trait Loci Mapping for Drought Tolerance in Ramie

Identifying QTL for DT is a potent way of developing drought-tolerant ramie cultivars (Liu et al., 2017). DS is a polygenic trait, and hundreds of genes control various responses to DS (Li et al., 2019). Identifying QTL leads to marker-assisted selection (MAS) to speed up breeding programs (Rai and Rai, 2020). Hundreds of QTL in ramie have been identified for various abiotic stresses and other important agronomic traits; however, no QTL has been cloned against DS tolerance (Li et al., 2019). In ramie, breeders have mapped the QTL for fiber yield, physiological traits, and other traits (Huang et al., 2021). Ramie is not a highly studied crop; therefore, evaluating ramie under DS and use of high-density markers would identify potent QTL for developing drought-tolerant cultivars (Liu et al., 2017). Therefore, it is important to expose the ramie genotypes to certain levels of DS and identify the putative QTL for DT which can be used to accelerate the MAS to develop tolerant cultivars. The evaluation of ramie populations in hydroponic conditions could be an effective way of screening against DS tolerance.

## Use of Speed Breeding Technique for Development of Drought-Resilient Cultivars

Speed breeding technique has gained world attraction. The University of Queensland scientists successfully grew wheat crop plants in space using artificial intelligence (AI). Recently, a scientist, Watson et al. (2018), developed a protocol for crops under the speed breeding system. This is one of the most potent ways of crop development to reduce time and cost (Watson et al., 2018). Plants were exposed to light for 22 h using several light resources. This procedure ensures an extended day length for crops and maximum light duration to speed up photosynthesis, leading to the quick development of flowers and seeds (Ghosh et al., 2018).

**FIGURE 5:**
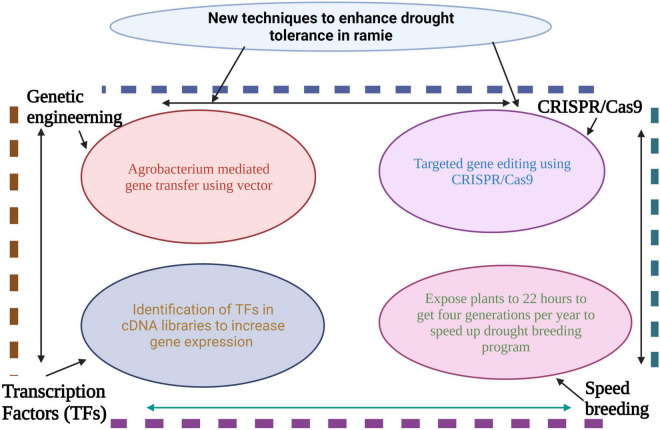
Use of several techniques to develop drought resilient ramie cultivars. Genetic engineering technique is used for gene transfer using *Agrobacterium* vector. CRISPR/Cas9 and transcriptome techniques can be widely used for enhancement of drought tolerance in ramie. Speed breeding can also be used for reduce the time period for development of tolerance ramie cultivars.

The speed breeding technique is being used to bring about a revolution in the agricultural sector and can be used to identify the genes, mapping population, and crossing of genotypes (Hickey et al., 2019). Speed breeding can be used to get four generations of any crop compared to conventional breeding (Watson et al., 2018). Speed breeding provides a quick, efficient, and reliable method of crop improvement in many sectors from phenomics to genomics. The integrated use of NGS and speed breeding can be helpful in the quick identification of novel genes, and development of drought tolerant crops (Razzaq et al., 2019). Therefore, speed breeding will (Figure 5) offer an exciting method for drought improvement in crops by mixing it with the next-generation OMICS techniques (transcriptomics, proteomics and metabolomics) to quicken crop breeding agendas (Razzaq et al., 2019). The above findings showed that speed breeding could also be used to develop drought-tolerant cultivars in ramie (Figure 5). Currently, no study has been reported on speed breeding in ramie; however, this technique offers good potential for breeding drought-resilient ramie crops.

## Engineering Drought Tolerance in Ramie Using CRISPR/Cas9

The CRISPR/Cas9 system is used on a large scale to develop immunity in crops against foreign invaders (Marraffini and Sontheimer, 2010). The CRISPR/Cas9 system is based on two components, single guided RNA (sgRNA), and Cas9 protein. sgRNA recognizes the target, and Cas9 protein cuts the targeted gene within the genome (Hsu et al., 2014). The binding of sgRNA and Cas9 to the region and cutting of the gene depends on the protospacer adjacent motif (PAM) sequence present in the downstream area of the targeted end. Hence, the use of different components makes it an easy and simple way of genome editing and expands its range behind biological boundaries. The improvement of targeted traits using CRISPR/Cas9 is highly successful, but particular PAM types limit its use to potential sites.

For this reason, multiple Cas9 variants have been developed with different PAM functions to edit the gene behind the biological areas (Wrighton, 2018). A recent example of using these variants can be seen in rice (Hu et al., 2018). The horizon of genome editing has been broadened by utilizing the type V CRISPR/Cas12 system (Zetsche et al., 2015). Recently, the use of dead Cas9 and Cas12 expanded the scope of CRISPR/Cas9 to develop mutant libraries, gene editing, and the generation of epigenetic variations (Wang et al., 2021). Readers can find multiple review articles presenting details of the structure and use of CRISPR/Cas9 in multiple crops (Jaganathan et al., 2018; Zhang et al., 2019).

Nowadays, CRISPR/Cas9 has been used on a large scale due to its broad adoption in editing crop genomes (Wang et al., 2016). This mechanism involves the sgRNA and Cas9 protein complex, which cause double-stranded breaks (DSBs) in DNA strands. These breaks are repaired by various mechanisms like homology direct repair (HDR) and non-homologous end joining (NHEJ) (Pawluk et al., 2018). CRISPR/Cas9 is now being used to achieve multiple stress resistance in crops to ensure global food security (Jain, 2015). These novel molecular techniques have highlighted the different factors which regulate plant response to drought (Farooq et al., 2021). Under DS, ABA acts to close the stomate and increased gene expression in order to prevent harmful effects (Sreenivasulu et al., 2012). In crops like ramie, ABA-related genes improved DT (An et al., 2015a). The bZIP is a significant TF in controlling DT because it promotes antioxidant mechanisms in crops and activates genes in ABA-related pathways (Huang et al., 2016a). With time, genome editing techniques are gaining more and more attention and providing new opportunities to improve desired traits of economic importance. With the development of gene editing tools, transcription activator-like effector nucleases (TALENs) and meganucleases (MNs), plant breeders can now target any gene of interest to bring about targeted variations. These techniques have several drawbacks like they need a complex phase that includes editing of protein. Compared to these techniques, CRISPR/Cas9 offers a simple and effective gene editing procedure. The Cas9 and several sgRNA target several sites in genomes (Feng et al., 2013). With the passage of time, the identification of several Cas9 enzymes from bacteria have enhanced the accuracy and effectiveness of genome editing (Ma et al., 2015). In ramie, no study has been reported on CRISPR/Cas9 gene editing; however, we believe that the future use of this technique will bring fruitful results in the development of drought-tolerant cultivars. Ramie is a significant fiber crop and use of CRISPR/Cas9 will be useful in developing drought tolerant cultivars. Different types of ramie cultivars including cultivated and wild relatives can be targeted by CRISPR/Cas9 to bring novel variations for DT. Ramie offers the wide use of CRISPR/Cas9 because of its great economic importance and potential threats by DS. Hence, CRISPR/Cas9 applications would open new doors for future research studies.

## Genetic Engineering for Drought Stress Tolerance in Ramie

Agricultural food production has been increased to a significant level; however, growing food demand for future populations is still a big challenge (Milestad, 2022; Panda et al., 2022; Wang et al., 2022). Several abiotic stresses affect crop growth and production and increase food security risks (Habibpourmehraban, 2022). Drought is one of the severe abiotic stresses which has a huge impact on the global food supply chain (Malik et al., 2021; De Freitas et al., 2022; Muhammad et al., 2022). DT is an important trait that plants need to survive under DS (Yadav et al., 2021; Batool et al., 2022b; Sakoda et al., 2022). The use of proper breeding methods makes it easy for breeders to maintain crop production under DS (Fahad et al., 2021b). Nowadays, traditional breeding has been replaced by molecular breeding to save time and cost. Although transgenic approaches have been successfully applied in many crops, the first successful application of GE in ramie was reported in 2014. The stress-responsive gene *SNACI* was first transferred into ramie using the *Agrobacterium* transformation method. The *Agrobacterium tumefaciens* strain, LBA4404 was used for successful transformation of genes (Figure 5). The gene overexpressed and increased DT in ramie plants. The results were collected using the data of the wild-type and engineered plants. Furthermore, the *SNACI* gene also improved DT at the rapid growth stage. Transgenic plants showed higher water content and photosynthetic rates than wild types. These results proved that GE techniques can improve DT in ramie (An et al., 2015b).

## Role of Transcription Factors in Drought Stress Tolerance in Plants

The TFs are often defined as the proteins resulting from transcription of DNA into RNA during the transcription process (Wani et al., 2021; Wu et al., 2022). TFs comprise a broad range of proteins, and they are responsible for the inhibition and regulation of genes for a particular function. They are described by the existence of DNA-binding domains that allow them to attach to a specific type of DNA called a promoter. These TFs can initiate transcription by connecting with a sequence of DNA promoters near the site of transcription. Table 2 shows different TFs for DT in various crops. The TFs, *OsMFTI, TaSNAC4-3A*, and *ZmNAC49* conferred DT in rice (*Oryza sativa*), wheat (*Triticum aestivum*), and maize (*Zea mays*) (Chen et al., 2021; Mei et al., 2021; Xiang et al., 2021). TFs are isolated from plants using various methods. Recently, the use of the whole genome sequencing technique has generated a large number of sequences and many databases are produced and maintained regarding TFs. The prominent example of these databases includes the TF library (Mitsuda et al., 2010) and PKU Yale (Gong et al., 2004). The clones of TFs in cloning vectors are also maintained by these libraries to facilitate researchers in crosschecking their TFs and selecting them for their specific role in stress tolérance in plants (Wehner et al., 2011). A high-quality web-scale tool like PlantTF-cat is often utilized to identify and categorize the particular TF (Dai et al., 2013). Gene regulation at a fixed time, cell, and TF abundance is a basic necessity for the sustainable development of cells. The gene expression process mainly involves the resequencing of DNA and energy usage.

**TABLE 2:** Different TFs for drought tolerance in economically essential crops.

Crop	Genes	References
Rice	*OsMFT1*	Chen et al., 2021
Wheat	*TaSNAC4-3A*	Mei et al., 2021
Maize	*ZmNAC49*	Xiang et al., 2021
Cotton	*GhWOX4*	Sajjad et al., 2021
Sugarcane	*AtBBX29*	Mbambalala et al., 2021
*Arabidopsis*	*CaNAC46*	Ma et al., 2021
Soybean	*GmMYB14*	Chen et al., 2021
Barley	*HvMYB1*	Alexander et al., 2019

The expression of stress-related genes in the cell requires a continuous flow of energy for transcription and unfolding DNA strands. The induction and suppression of downstream genes is initiated by TF when necessary. This TF plays a beneficial role in plant stress responses, as evidenced by many published reports. For instance, in the WRKY TF gene, the promoter region has a W box to which the gene binds to its promoter during insect attack and provides resistance to plants (Birkenbihl et al., 2012). The abiotic stress resistance triggered the ABA accumulation, which initiated the miRNA159 accumulation. This process degrades the MYB33 and implicates stress susceptibility (Reyes and Chua, 2007). The biotic stress signals aid the transportation of *bZIP28* from the endoplasm to Golgi, stimulating the genes expressed in the nucleus (Liu and Howell, 2010). After the stress withdrawal, plants need to stop the activity of TFs to avoid the overuse of energy (Sadhukhan et al., 2014). In the subsequent review, we have described the significant TFs found in ramie, with their gene regulatory system, which played a part in DT.

## Identification of Drought-Responsive Transcription Factors in Ramie

Plants have developed a complex network to deal with severe environmental stresses Rasheed et al., 2020a,b,c,d, 2021a,b,c,d; Fahad et al., 2021a,b). Different genes are involved in regulating DT in crops (Rasheed et al., 2020c, 2021a). Five leading gene families have gained widespread attention because of their crucial role in DT (Gahlaut et al., 2016). However, in the case of ramie, we have briefly discussed the role of different TFs involved in DT. TFs perform a reversible phosphorylation role and make a complex network. Various TFs were identified in ramie in a previous study based on the data collected under control and DS. Tags were generated from libraries and aligned with ramie TFs to study their role, and 22,826 genes were compared by using these tags. Comparison of the expression level of genes among the DS and control ramie on the basis of variation in tags frequencies in libraries showed that there are about 1516 DT genes, and 24 of them are TFs. The Unigene19721 encoding DELLA protein was found up-regulated under water stress, and it is a negative regulator of GAs and involved in growth inhibition of ramie under DS. The change in expression of these TFs was further validated under DS conditions. TFs were chosen from well-watered and drought-stressed ramie, and their expression level was further confirmed by real-time quantitative polymerase chain reaction (PCR). Results concluded that out of the 24 TFs, 12 were involved in response to DS (Liu et al., 2013c).

Drought regulatory mechanism is still unknown in ramie. Polyethylene glycol (PEG) treatment is the most common and widely adopted way to impose DS in plants. An et al. (2015a) made a cDNA library collection and studied the transcriptome analysis of ramie cultivars subjected to DS imposed by PEG treatment. The study involved illumining paired-end sequencing, which produced over 170 millions sequence reads. Roots and leaves were subjected to PEG treatment for 24 and 72 h (L1, L2, L3, and L4). Results showed that 16,798 genes were expressed; out of them, 8627 expressed in roots and 9281 were expressed in leaves. A total of 25 TFs were involved in the DS response in ramie. Further analysis of these TFs could provide the practical basis for understanding the DS tolerance mechanism in ramie. There are only a few TFs still known, and it is a significant obstacle in understating the mechanism of DS tolerance in ramie (Zheng et al., 2016). To uncover more DT genes and TFs, a total of 179 genes with length reading frames from bZIP and COL families were gained by searching against the ramie TFs. Studying the expression pattern demonstrated that these genes showed a higher expression pattern in stem xylem and a lower expression pattern in other tissues. A total of 96 genes were involved in DT. They have concluded that these TFs play a crucial role in stress tolerance in ramie. These results will help unfold the stress-responsive mechanism in ramie (Zheng et al., 2016).

**FIGURE 6:**
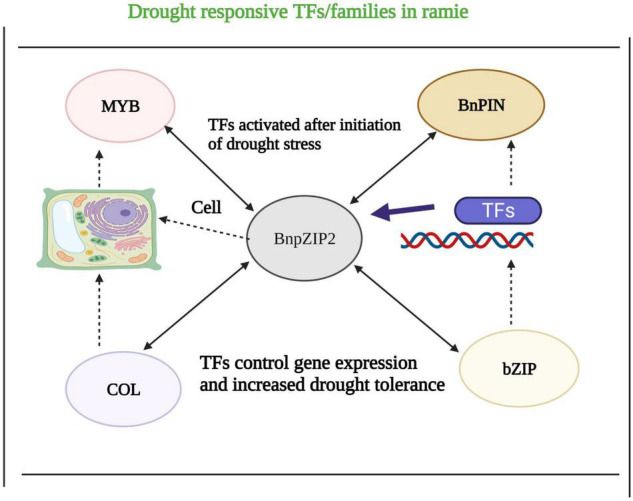
Role of different TFs in ramie crop under severe drought stress (DS). TFs have a key role in drought tolerance. MYB, COL, bZIP, and BnPIN showed enhance tolerance to DS. These TFs regulate gene expression and enable plant to withstand with drought episodes.

In another study, qRT-PCR was used to study the function of two gene families. Different analysis like phylogenetic relationship, intron/exon, and expression patterns were studied in tissues. The expression pattern was studied in response to DS induced by PEG treatment. The BnPIN (Table 3) gene was upregulated because of the DS treatment. These studies provided new insights for further analysis of the biological roles of ramie against environmental stresses (Bao et al., 2019). Huang et al. (2016a) cloned a novel bZIP gene known as *BnbZIP3* from ramie plants based on their Unigene6582 sequence in TFs using the CDNA amplification technique and PCR (Figure 6). The study results suggested that *BnbZIP3* shared higher sequence identities to bZIP factors from other ramie plants. The fusion of gene with ECFP was done, and it showed the subcellular localization of protein. Different transcripts of ramie were found in ramie plants. The induction of DS increased the expression pattern of *BnbZIP3*. The *cis*-acting elements of this gene are involved in multiple stresses response mechanisms. Results showed that the same gene inhibited *Arabidopsis* growth under normal circumstances and increased dehydration under stress conditions. This gene may be helpful in developing drought-tolerant ramie cultivars (Huang et al., 2016a).

**TABLE 3:** List of different transcription factors/genes involved in drought stress (DS) tolerance in ramie.

Families	Name of gene	Number of genes	References
MYB, bZIP	*BnMYB01–BnMYB67*	1	Zheng et al., 2016
bHLH	*Unigene4099*	1	Liu et al., 2013c
NAC	*Comp56509*	9	An et al., 2015a
BnPIN	*BnPIN3*	1	Bao et al., 2019
bZIP	*BnbZIP3*	1	Huang et al., 2016a
bZIP	*BnbZIP2*	1	Huang et al., 2016b

Another ramie bZIP gene (Table 3 and Figure 6), that has a crucial role in stress response was cloned from ramie plants. *BnbZIP2* transcripts are found in many tissues in ramie plants. The gene expression was induced by DS. *Cis*-acting analysis of *BnbZIP2* showed that this gene involved multiple stress response mechanisms in ramie. Transgenic *Arabidopsis* plants with overexpression of this gene, *BnbZIP2* showed high sensitivity to DS whereas high tolerance to salinity stress than wild type at seed germination stage. This gene may act as a positive regulator of DS tolerance in ramie (Huang et al., 2016b). These studies analyzed the role of different TFs in ramie under DS. Ramie is not a highly studied crop regarding its genetics and wide-scale adaptation. Further studies focusing on identifying and analyzing each TF gene will speed up the study of the genetic mechanisms of ramie’s tolerance to DS. The development of drought-tolerant ramie cultivars will boost the industrial use of ramie crop (Bao et al., 2019).

## Conclusion and Future Recommendations

Crop growth and production have been highly affected by severe environmental constraints; one of them is DS which causes a significant loss in yield and quality of agriculturally important crops. The simultaneous incidence of drought induced osmotic and oxidative stress occurs on a common basis. Plants balance these dangers by accumulating compatible solutes in cells and detoxifying ROS, and modifying the actions of antioxidant enzymes. Plants have evolved various morphological, biochemical, and molecular mechanisms to cope with DS episodes. Complete knowledge of DT at the molecular level would help scientists identify and clone the regulatory elements used in molecular breeding. CRISPR/Cas9 mediated genome editing of ramie is a novel and powerful way to edit and manipulate any gene across biological boundaries. The speed breeding, transgenic breeding, and identification of TFs are highly effective and reliable techniques to develop drought tolerant ramie cultivars. More investigation into the role of plant growth-promoting hormones is necessary to help understand plant strategies against stress. A more inclusive image of the upregulated genes in response to DS can be recognized, categorized, and genetic alterations can be promising for crop development schemes. As discussed in this review, different plant mineral nutrients like NPK and micronutrients like Ni, Zn, Co, B, and Se can improve the growth and development of plants, both under usual and stressed situations, by preventing the oxidation of polyunsaturated fatty acids (PUFAs), thus preventing membrane leakage and unnecessary development of free radicals. Due to increased plant nutrition under DS, the resistance level would increase, and the plant can maintain its growth and yield. There are few studies published which deal with the mechanisms and improvement of DT in ramie. There are many questions which are not answered. Complete genetic control of DT is not understood and needs further study. Hence, it is concluded that the use of certain molecular, as well as conventional approaches, are mandatory to improve the growth and yield of ramie under DS. Therefore, it would be better to test the genotypes against certain levels of DS under controlled conditions.

## Author Contributions

AR conceptualized and prepared the manuscript. YJ reviewed the manuscript, supervised the study, and provided the funding. MN, HJ, and YM provided the technical assistance. ANS, MUH, SFAG, MB, MTA, ARN, and SHQ reviewed the manuscript. All authors contributed to the article and approved the submitted version.

## Conflict of Interest

The authors declare that the research was conducted in the absence of any commercial or financial relationships that could be construed as a potential conflict of interest.

## Publisher’s Note

All claims expressed in this article are solely those of the authors and do not necessarily represent those of their affiliated organizations, or those of the publisher, the editors and the reviewers. Any product that may be evaluated in this article, or claim that may be made by its manufacturer, is not guaranteed or endorsed by the publisher.
